# Urine Metabolomics Study on Potential Hepatoxic Biomarkers Identification in Rats Induced by Aurantio-Obtusin

**DOI:** 10.3389/fphar.2020.01237

**Published:** 2020-08-12

**Authors:** Longlong Xu, Yuguang Wang, Zengchun Ma, Xianglin Tang, Yue Gao

**Affiliations:** ^1^ College of Life Science and Bioengineering, Beijing University of Technology, Beijing, China; ^2^ Department of Pharmaceutical Sciences, Beijing Institute of Radiation Medicine, Beijing, China

**Keywords:** aurantio-obtusin, liver injury, urine, metabolomics, ultraperformance liquid chromatography quadrupole time-of-flight mass spectrometry

## Abstract

Previous studies revealed the hepatotoxic effect of aurantio-obtusin on rats. The aim of this study was to identify potential biomarkers of urine caused by aurantio-obtusin. Sprague–Dawley (SD) rats with body weight of 0, 4, 40, and 200 mg/kg were orally given aurantio-obtusin for 28 days, and urine was collected for 24 h after the last administration. The urine metabolites in the aurantio-obtusin group and the control group were analyzed by ultraperformance liquid chromatography quadrupole time-of-flight mass spectrometry (UPLC-QTOF/MS). Twenty-three metabolites were identified as potential biomarkers, and 10 of them were up-regulated, including xanthosine, hippuric acid, 5-L-glutamyl-taurine, etc. The other 13 biomarkers were down-regulated, including thymidine, 3-methyldioxyindole, cholic acid, etc. The significant changes of these biomarkers indicated that purine metabolism, taurine and hypotaurine metabolism, primary bile acid biosynthesis, pyrimidine metabolism, and tryptophan metabolism played an important role in the hepatotoxicity of aurantio-obtusin in rats. In this paper, the safety and potential risk of aurantio-obtusin were studied for the first time by combining the toxicity of aurantio-obtusin with the results of urine metabolomics, which provided information for the mechanism of liver injury induced by aurantio-obtusin.

## Introduction

Anthraquinone, also known as 9,10-anthraquinone, belongs to polycyclic aromatic hydrocarbons. Anthraquinone and its derivatives are naturally found in many plants. They are important dietary components of human beings, not only in peas, cabbage, lettuce, beans, and other vegetables, but also the main active components of traditional Chinese medicines (TCMs) such as *Polygonum multiflorum* Thunb, *Rhubarb*, *Cassiae* semen, etc. (Li and Jiang, 2018). Anthraquinones have the medicinal value of anti-tumor, anti-bacterial, anti-inflammatory, anti-oxidation, anti-diabetes, and anti-hypertension (Hussain et al., 2015). In recent years, TCMs containing anthraquinones have been widely used in medicine and functional food. However, there are increasing reports of adverse reactions caused by taking TCMs containing anthraquinones (Malik and Müller, 2016). It was reported that some anthraquinone cathartics could cause hepatitis (Beuers et al., 1991; Seybold et al., 2004; Rabe et al., 2005; Bottenberg et al., 2007; Yang et al., 2010), but the evidence level of causal relationship was rarely defined (Teschke et al., 2013; Teschke and Eickhoff, 2015). As a result, people pay more and more attention to the safety of TCMs containing anthraquinones. Therefore, the safety evaluation of anthraquinones has become an urgent problem to be solved in TCMs and functional food industry. The report of liver injury caused by *Polygonum* *multiflorum* Thunb, a TCM for tonifying liver and kidney, has aroused widespread concern (Zhang et al., 2020). However, it was not clear about the components, biological characteristics, and mechanism of liver injury caused by *Polygonum multiflorum* Thunb (Li et al., 2016; Li et al., 2017). Most studies indicated that the hepatotoxicity of *Polygonum multiflorum* Thunb should be related to anthraquinones, especially emodin and its derivatives (Dong et al., 2016), but the effects of other anthraquinones on the liver still needed to be further investigated. Similarly, *Rhubarb* containing toxic anthraquinone components was considered to be hepatorenal and carcinogenic (Byeon et al., 2019). However, the toxicity of purified anthraquinone, especially for natural compounds, was limited. Available data showed that some anthraquinones were toxic at least.

*Cassiae* semen is a dry and mature seed of *Cassia obtusifolia* L. or *Cassia tora* L. It was firstly recorded in Shennong Materia Medica. As a kind of TCM with homology of medicine and food, it has the functions of reducing blood pressure, regulating serum lipid, protecting liver, etc. It is commonly used in the treatment of hyperlipidemia, hypertension, constipation, and other diseases (Dong et al., 2017). Aurantio-obtusin (**Supplementary Figure S1**) is a unique anthraquinone compound of *Cassiae* semen. It was firstly used as the quality standard of *Cassiae* semen in 2010 edition of Chinese Pharmacopoeia, and the content of aurantio-obtusin in *Cassiae* semen should not be less than 0.080%. At present, the research of aurantio-obtusin is mainly focused on its anti-inflammatory effect (Hou et al., 2018; Kwon et al., 2018), and there is little toxicological evaluation on aurantio-obtusin. Anthraquinones are the main active components of *Cassiae* semen, mainly including rhein, emodin, aloe-emodin, chrysophanol, physcion, chryso-obtusin, obtusifolin, obtusin, and aurantio-obtusin (Xie et al., 2019).

However, the effective components of TCMs are often toxic, so it is very important to study the toxicity of monomer of TCMs (Gao et al., 2019). Although there were many reports about different pharmacological effects of *Cassiae* semen extract, its toxicological effect was rarely reported. For example, previous studies showed that *Cassiae* semen extract could cause hepatocytes injury with cholestasis type liver damage. However, the specific components of drug-induced liver injury were not clear (Peng et al., 2016). Yang et al. indicated that the anthraquinones existing in *Cassiae* semen (obtusifolin, aurantio-obtusin, and obtusin), emodin, and rhein were the potential hepatotoxic phytochemicals in the aqueous extract (Yang et al., 2019). Considering the fact that there are lots of anthraquinones in *Cassiae* semen, it is speculated that *Cassiae* semen may have similar toxic reactions with *Polygonum multiflorum* Thunb and *Rhubarb*. However, it is not clear which component of anthraquinones is responsible for the toxicity. In order to identify its toxic component, aurantio-obtusin, a unique anthraquinone in *Cassiae* semen, was studied in this paper.

Pharmacometabolomics is an extremely sensitive and comprehensive method to study the low toxicity of drugs based on chemometrics. Administration of aurantio-obtusin, the degree of deviation of endogenous metabolites from normal physiological state is used to express the toxicity of aurantio-obtusin. As a means of toxicity evaluation, metabolomics has its unique advantages (Lao et al., 2009). It can find toxic substances faster and more accurately compared with traditional methods. In our previous study, we reported that aurantio-obtusin could cause liver injury in rats from the perspective of serum metabolomics combined with biochemical, hematological, and pathological indicators. We also found metabolic alterations in liver injury, including metabolism of amino acids (e.g., vanylglycol, phosphopantothenoylcysteine, and dehydroalanine) and lipids (e.g., cholic acid, taurodeoxycholic acid, and phosphatidylcholines) in a serum metabolomics study (Xu et al., 2019). At present, there is no metabolomics applied to the study of urinary metabolism after aurantio-obtusin administration. Therefore, we established the method of urinary metabolomics and determined the biomarkers of aurantio-obtusin for the early diagnosis of toxicity in this study.

## Materials and Methods

### Drugs, Reagents, and Instruments

Aurantio-obtusin (purity 98%) was purchased from Beijing Saibaicao Technology Co., Ltd. (Beijing, China). Acetonitrile (HPLC-grade) was acquired from Thermo Fisher Scientific Co., Ltd. (Shanghai, China). Formic acid (HPLC-grade) was obtained from MREDA Technology, Co., Ltd. (MREDA, USA). The ultra-pure water was prepared by the Milli-Q system (Millipore, Bedford, MA, USA). Carboxymethyl cellulose sodium salt (CMC-Na) was provided from Sinopharm Chemical Reagent Co., Ltd. (Beijing, China). All other reagents were analytical grade. Analysis of urine was performed on an ACQUITY UPLC system (Waters Technologies, USA) equipped with a Synapt G2-Si mass spectrometer.

### Animal Administration

Male Sprague-Dawley rats [special pathogen free, spruce-pine-fir (SPF) grade], weighing 140–180 g, were purchased from Beijing Vital River Laboratory Animal Technology Co., Ltd. with license number of SCXK (Beijing) 2016-0006, and kept in the experimental animal center of the Academy of Military Medical Sciences. After 1 week of adaptation, eight rats in each group were randomly divided into the control group and low, medium, and high dose group of aurantio-obtusin (equivalent to 1, 10, and 50 times of adult clinical dose respectively). Aurantio-obtusin was dispersed in 0.5% CMC-Na aqueous solution, and aurantio-obtusin was given by gavage in the low, medium, and high dose groups at doses of 4, 40, and 200 mg/kg, respectively. Meanwhile, the control group was given the same volume of 0.5% CMC-Na solution every day. All groups were administrated once a day for 28 days.

### Sample Collection and Pretreatment

After the last gavage, all rats were put into the metabolic cage, fasted but drank freely. The urine of single animal was collected for 24 h, centrifuged at 12,000 r/min for 10 min, and stored in a refrigerator at −80°C. Before the sample was tested, the urine sample was taken out and thawed at room temperature. After centrifugation at 12,000 r/min for 10 min, the supernatant was filtered with 0.22 μm microporous membrane for UPLC-QTOF/MS determination.

### Preparation of Quality Control Samples

We took 100 μl urine to be tested from all the urine samples and processed them according to the same pretreatment method in 2.3 to obtain the quality control (QC). In order to ensure the stability of the whole analytical system, QC was used to verify the method in this experiment. Before analyzing the samples, QC samples were run five times to balance the system. After every eight samples a QC sample was analyzed to ensure the stability and repeatability of analysis.

### Ultraperformance Liquid Chromatography Quadrupole Time-of-Flight Mass Spectrometry Conditions

The liquid chromatogram (LC) conditions were analyzed with an ACQUITY UPLC HSS T3 column (2.1 mm×100 mm, 1.8 μm). The flow rate was controlled at 0.5 ml/min, and the temperature of the automatic injector and the column was controlled at 4 and 40°C, respectively. The injection volume of all samples was 5 μl. Mobile phase A and B were water and acetonitrile (ACN), respectively. The two phases contained 0.1% formic acid (FA). The linear gradient elution procedure was shown in **Supplementary Table S1**. The condition of mass spectrometry (MS) analysis was electrospray ionization (ESI) source ionization in positive and negative ion mode respectively, and the scanning mode adoped centroid and continuum mode. The optimum conditions were: capillary voltage 3.0 kv, flow rate 850 L/h, source temperature 100°C, desolvent gas temperature 450°C. In the calibration mode, sodium formate solution was used for automatic calibration from *m/z* 50 to 1,000, and leucine enkephalin was used for real-time and accurate mass calibration.

### Data Processing and Statistical Analysis

The samples were detected by UPLC-QTOF/MS to obtain the total ion chromatogram. The MS raw data were converted into mzXML format using MS Convert software, and XCMS (www.bioconductor.org/) was used to extract the peak data, peak denoise, peak matching, peak alignment, and export. Then the pre-processed data were imported to the SIMCA-P 11.0 software (Umetrics AB, Umea, Sweden). The methods of data analysis were unsupervised pattern recognition (principal component analysis, PCA) and supervised pattern recognition (partial least squares discriminant analysis, PLS-DA). The score plot obtained by the orthogonal partial least squares discriminant analysis (orthogonal PLS-DA, OPLS-DA) could visually observe the distribution and difference of urine components between the control group and the high dose aurantio-obtusin group. The effective signals were the variables whose variable importance in the projection (VIP) was more than 1 and which had significant difference between the control group and the aurantio-obtusin group at 95% confidence level. All the statistical analyses were performed by SPSS Statistics ver18.0. The potential biomarkers were identified by searching the Human Metabolome Database (HMDB) and Kyoto Encyclopedia of Genes and Genomes (KEGG) online databases, comparing the mass to nucleus ratio and mass spectrum. According to the existing standards in our laboratory, we compared the MS/MS patterns of resultant metabolites. Finally, metabolic pathways of biomarkers in urine were analyzed by using MetaboAnalyst 4.0 (http://www.metaboanalyst.ca).

## Results

### Quality Control in Ultraperformance Liquid Chromatography Quadrupole Time-of-Flight Mass Spectrometry Analysis

In order to ensure the quality and reliability of the final collected data, it is necessary to investigate the stability of the method and to carry out PCA analysis on the data collected from QC samples. Theoretically, the QC samples should be exactly identical. In fact, there will be errors in the detection and analysis process, and differences in the QC samples. The QC samples were densely distributed in PCA analysis (**Supplementary Figure S2**). The point outside the circle indicated that there was a big difference between the metabolic spectrum of this sample in low-dose group and that of all other analysis samples (**Supplementary Figure S2A**). Generally, there will be a separate group point, which is a normal phenomenon. Moreover, this outlier was around the 95% confidence interval, not too far away. In addition, five ion signals were randomly selected to analyze their retention time and peak area in positive and negative ion modes. The relative standard deviations (RSD%) of retention time were 0~0.28 and 0% in positive and negative ion modes, respectively. The RSD% of the peak area were 0.78~7.67 and 1.42~7.07%, respectively (**Supplementary Table S2**). The results showed that the method had good repeatability, high precision, and could be used for batch metabolomics analysis.

### Ultraperformance Liquid Chromatography Quadrupole Time-of-Flight Mass Spectrometry Fingerprinting Analysis

**Figure 1:**
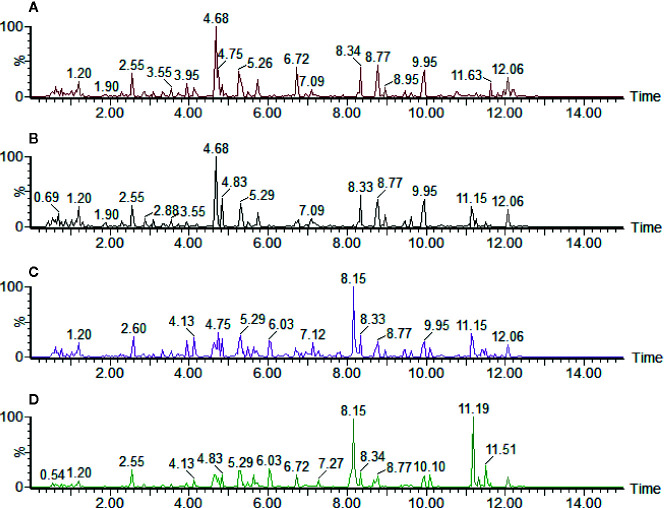
The ultraperformance liquid chromatography quadrupole time-of-flight mass spectrometry (UPLC-QTOF/MS) base peak intensity (BPI) profile of **(A)** control group, **(B)** low dosage group, **(C)** medium dosage group, and **(D)** high dosage group urinary samples on the 28th day in positive electrospray ionization (ESI) mode. Time unit is minute.

The typical positive and negative base peak intensity (BPI) chromatograms of urine samples were shown in **Figure 1** and **Supplementary Figure S3**, respectively. According to the BPI chromatogram, there were significant differences in the peak height and peak area of multiple peaks with the same retention time between the control group and the low, medium, and high dose groups of aurantio-obtusin. It was suggested that different doses of aurantio-obtusin could change the contents of some small molecular metabolites in rats. In addition, there were significant differences in the positive and negative ionization patterns of BPI chromatogram. Considering that different substances have different properties, the positive and negative ionization modes were used to detect different substances, making the detection of small molecular peak more comprehensively. We extracted 23 biomarkers with significant changes in the positive and negative ion mode, as shown in the **Supplementary Figure S4**.

### Multivariate Statistical Analysis

In order to observe the aggregation and dispersion of the samples, the PCA, PLS-DA, and OPLS-DA were used to establish the metabolomics model of the control and different doses of aurantio-obtusin groups after data preprocessing.

**Figure 2:**
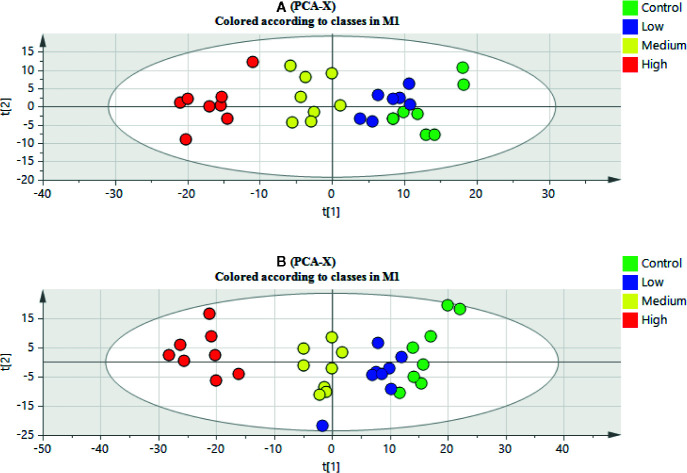
Principal component analysis (PCA) scores plot of urinary samples collected from control group, low dosage group, medium dosage group, and high dosage group in positive electrospray ionization (ESI) mode **(A)** and negative ESI mode **(B)**.

The clustering and changing trend of each sample could be intuitively displayed on the PCA score plot (**Figure 2**). The control group and the aurantio-obtusin groups were obviously separated. Meanwhile, the PLS-DA was used to establish the metabolomics model of the aurantio-obtusin groups and the control group in rat urine samples (**Supplementary Figure S5**). The changes in urine metabolites were distinct at different dosages. The samples containing different doses of aurantio-obtusin were separated from the control samples, showing a very clear line of motion. Previous studies indicated that the dose-dependent hepatotoxicity correlated with the dosage of aurantio-obtusin (**Supplementary Figure S6**). The degree of deviation of the urine samples was positively correlated with the dose of aurantio-obtusin. The high dose group deviated most from the other three groups, which indicated that aurantio-obtusin altered the normal physiological state of rats *in vivo*.

**Figure 3:**
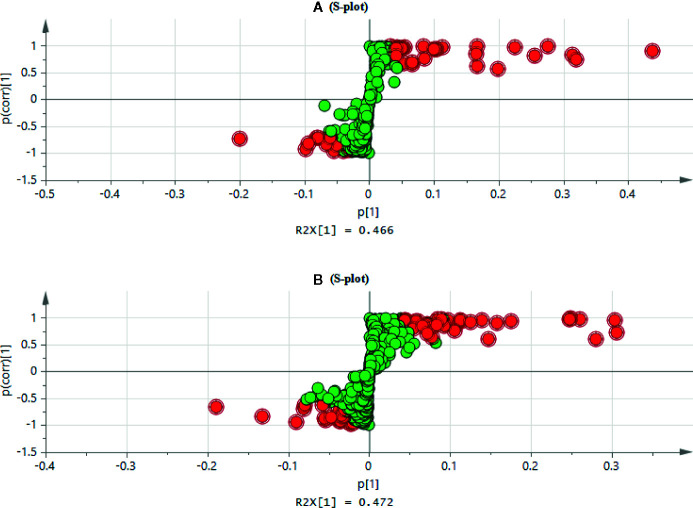
S-plot of the control and high dosage group in positive **(A)** and negative **(B)** electrospray ionization (ESI) mode.

The high dose group revealed a significant change of metabolites in comparison to the control group, so the OPLS-DA was used to monitor the changes of metabolites in the high dose group and the control group (**Supplementary Figure S7**). The OPLS-DA score plot showed that the separation between the control group and the high dose group of aurantio-obtusin was significant, indicating that the metabolite content of the high dose group of aurantio-obtusin was different from that of the control group. R2X (cum) and Q2 (cum) parameters were used to evaluate these models (**Supplementary Table S3**), which showed that the models of PCA, PLS-DA, and OPLS-DA had good fitting and prediction ability. Permutation tests (**Supplementary Figure S8**) with 200 iterations showed that the model was not over-fitted. As a visualization method, the S-plot was also used to select potential biomarkers (**Figure 3**). The distance between the ion and the origin represented the contribution to different clusters on OPLS-DA. In the S-plot, the farther the metabolite points were plotted on the X and Y axes, which contributed greatly to the differences between the control group and high dose group. Therefore, we chosed the metabolites far away from the origin as biomarkers of aurantio-obtusin.

### Biomarker Discovery and Identification

In this experiment, the urine samples of rats in the control group and high dose group were analyzed by the OPLS-DA. According to the S-plot, the difference variables causing the above separation were found. The importance of variables to classification was measured by the VIP value, and the variables were further screened according to VIP. The difference variables satisfying VIP > 1.0 and *P < 0.05* were screened out. The MS/MS spectra of authentic standards were obtained and used to confirm these identifications. An example was an ion of *m/z* = 178.0510 found to be elevated in the urine of aurantio-obtusin dosed rats where the postulated identification of the compound as hippuric acid was confirmed using the standard (**Supplementary Figure S8**). Finally, the potential biomarkers were identified by combining HMDB, KEGG, and other databases (**Supplementary Table S4**).

### Metabolic Pathway Analysis

**Figure 4:**
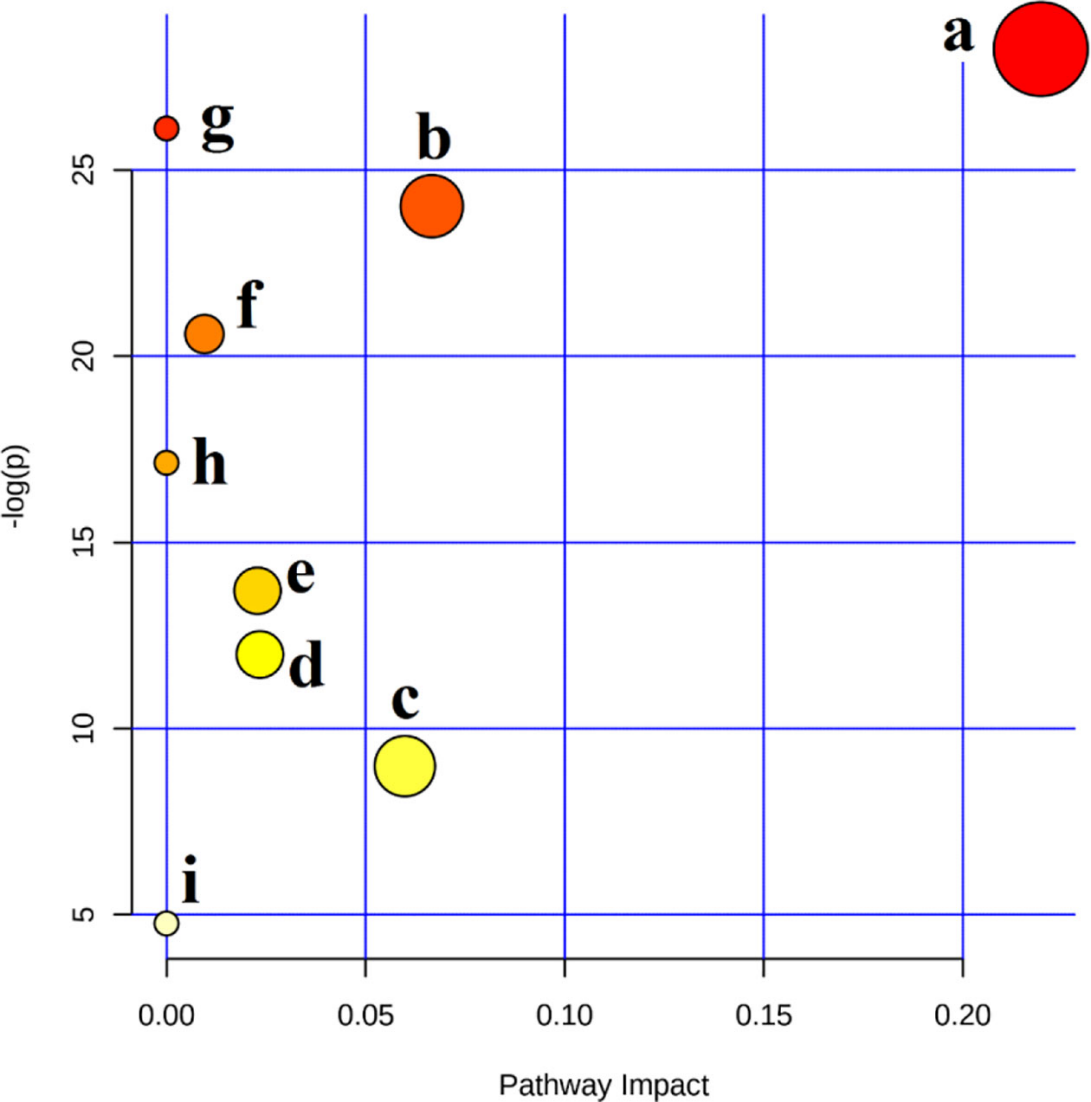
Summary of pathways analysis with MetaboAnalyst 4.0. (A) Glyoxylate and dicarboxylate metabolism; (B) glycine, serine, and threonine metabolism; (C) pyrimidine metabolism; (D) arginine and proline metabolism; (E) primary bile acid biosynthesis; (F) purine metabolism; (G) phenylalanine metabolism; (H) taurine and hypotaurine metabolism; (I) tryptophan metabolism.

**Figure 5:**
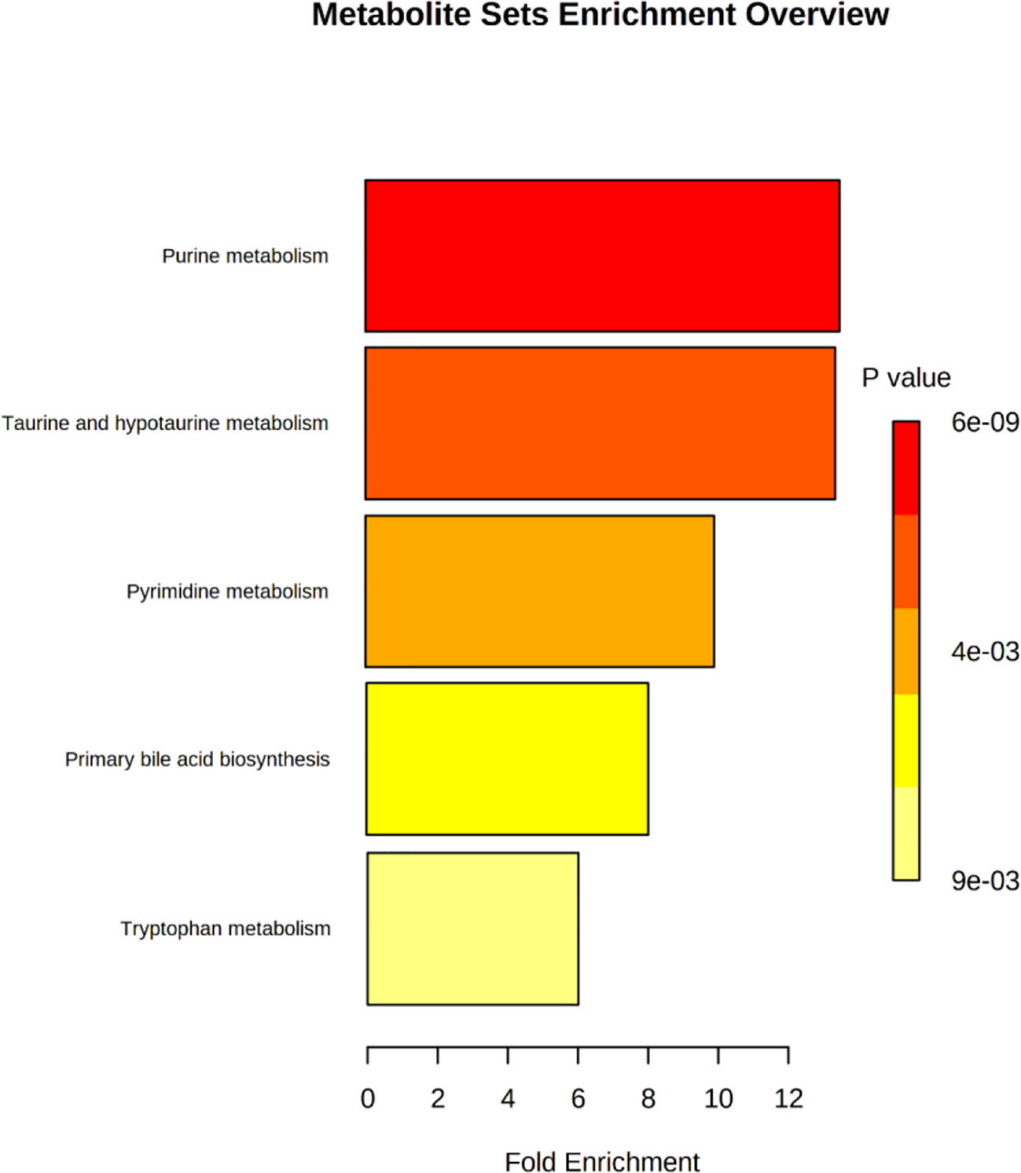
Metabolite sets enrichment analysis of potential biomarkers related to liver injury in rats treated with aurantio-obtusin.

In order to further explore the hepatotoxic mechanism of aurantio-obtusin, the different metabolites in the urine of rats in the control group and high dose group of aurantio-obtusin were substituted into the MetaboAnalyst 4.0 data processing platform for metabolic path analysis, as shown in **Figure 4**. The results showed that the six metabolic pathways disturbed by aurantio-obtusin were glyoxylate and dicarboxylate metabolism, glycine, serine and threonine metabolism, pyrimidine metabolism, arginine and proline metabolism, primary bile acid biosynthesis, and purine metabolism (**Supplementary Table S5**). Then I would like to collect the metabolites with the most significant fold change to discriminate the control and the high dose group and to see in which pathway these metabolites were involved. I did the metabolite set enrichment analysis (**Figure 5**).

### Correlation and Heatmap Analysis of Biomarkers

**Figure 6:**
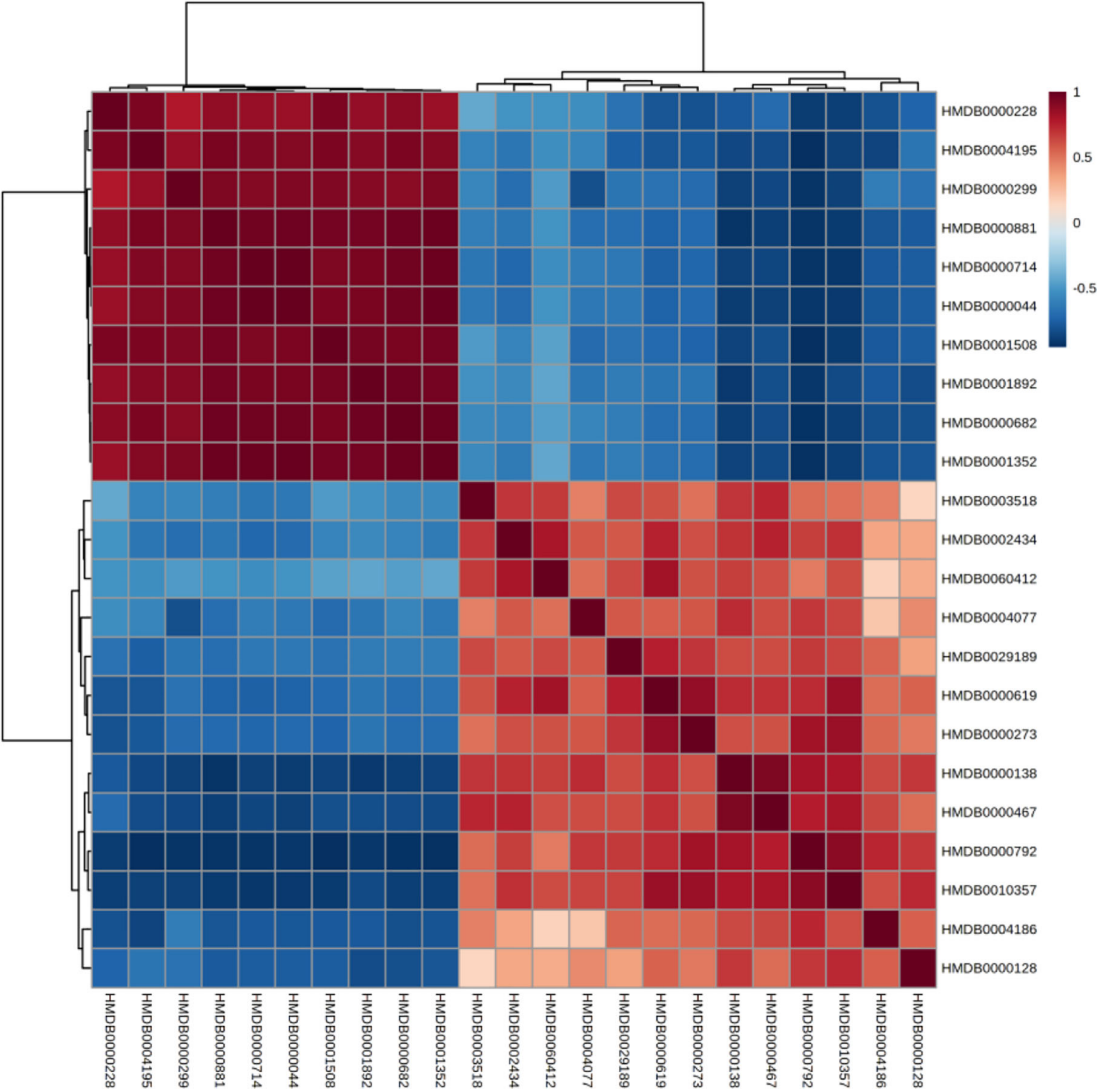
Correlation of differential urinary metabolites after administration of aurantio-obtusin.

**Figure 7:**
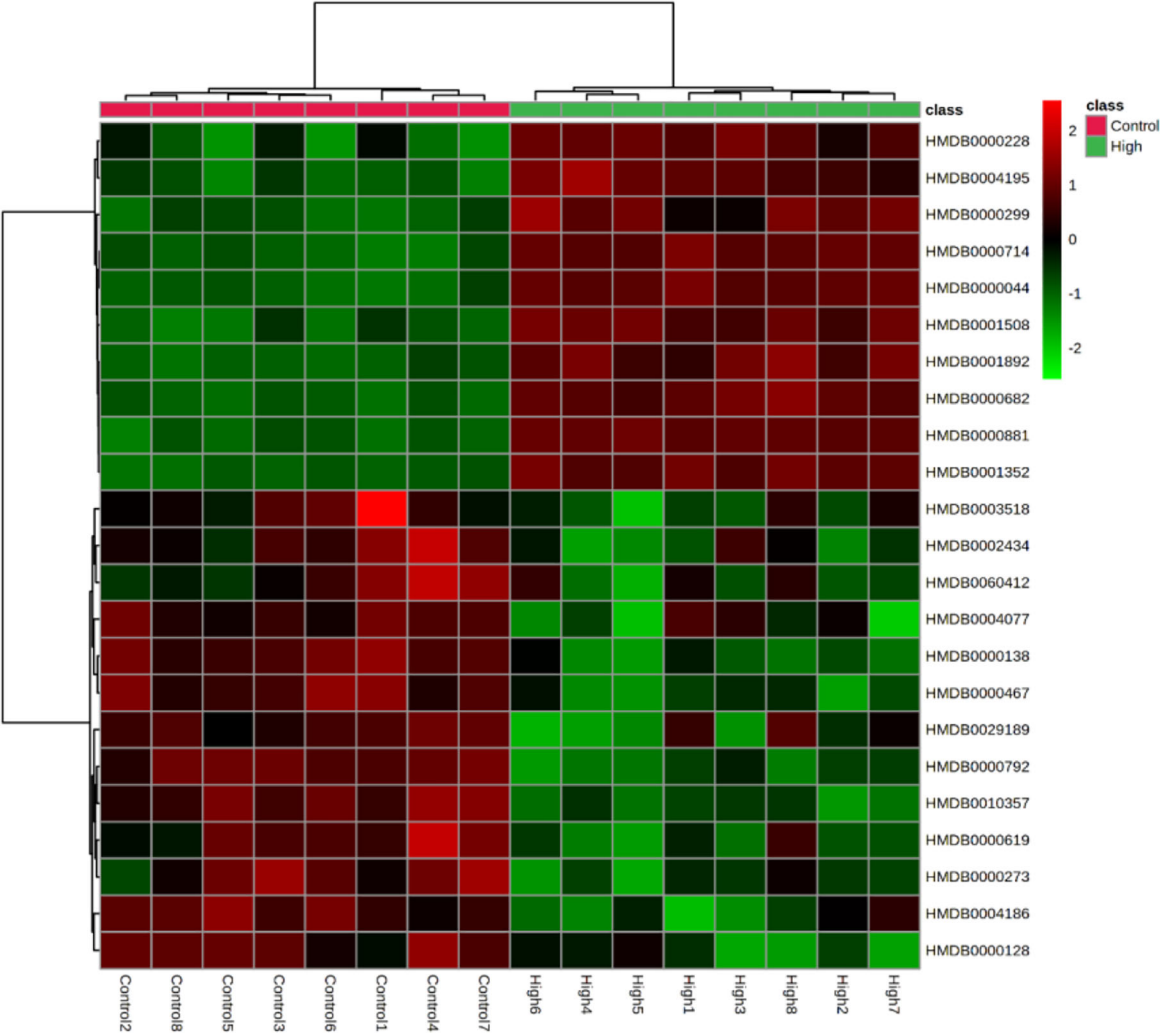
Heatmaps, generated by hierarchical Pearson clustering, of selected urine metabolites, significant between the control and high dose treated group by using Student’s t-test, *p-value < 0.05*.

The correlation between different compounds was shown in **Figure 6**. Red (value = 1) indicates high correlation between compounds, light red (value = 0.5) indicates medium correlation, while the darker blue indicates low correlation. Most of the small molecular compounds in the sample had a strong correlation. They had similar metabolic pathways and related biological effects. The specific functions of the key compounds would be explained in the *Discussion* section. To further understand metabolic differences between high dose group and the control group in the 28th day, the metabolites were visualized in a clustering heatmap (**Figure 7**), which revealed directly the variation of each metabolite in each rat. The concentrations of metabolites were shown in red to green gradients. Red represents high abundance metabolites. Green represents low abundance metabolites, and black represents medium abundance metabolites. The trend of metabolites in the control group and high dose aurantio-obtusin group could be easily observed from the heatmap. In addition, the relative concentrations of 23 metabolites were shown in **Supplementary Figure S10**. Compared with the control group, 3-methyldioxyindole, sebacic acid, 4,6-dihydroxyquinoline, guanidoacetic acid, nutriacholic acid, 6-methylmercaptopurine, cholic acid, thymidine, tetrahydroaldosterone-3-glucuronide, hydroquinone, 5-hydroxysebacate, homocitric acid, and glycocholic acid were significantly decreased, while deoxyadenosine diphosphate (dADP), hippuric acid, xanthurenic acid, xanthosine, ascorbic acid, 5-L-glutamyl-taurine, phenol, indoxyl sulfate, hydroxypyruvic acid, and menadione were significantly increased in the high group.

## Discussion

**Figure 8:**
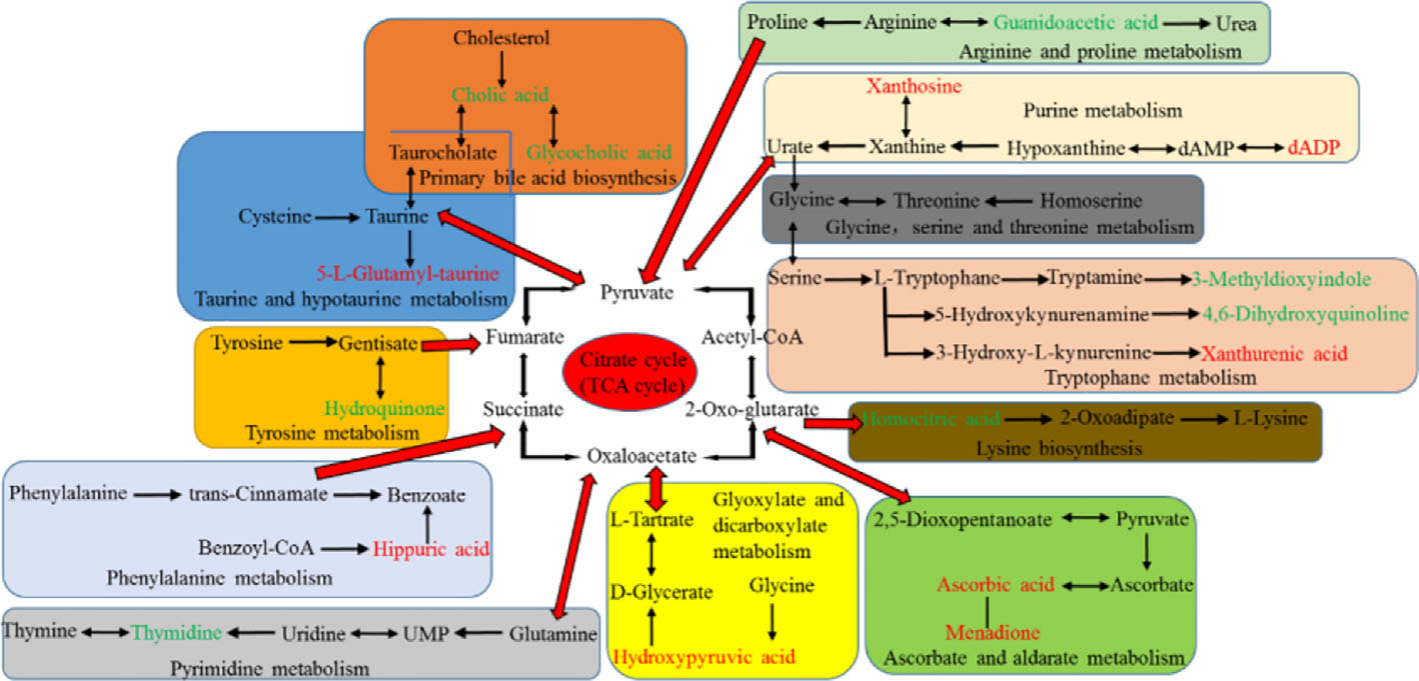
Correlation pathways of all the potential biomarkers in response to aurantio-obtusin. The metabolites colored with green and red show a significant decrease and increase in aurantio-obtusin group compared to control group, respectively.

The potential hepatotoxicity of aurantio-obtusin was determined on the basis of routine biochemical analysis and histopathological observation, and the serum metabolomics of aurantio-obtusin was studied to find the related serum metabolites. An important advantage of urine metabolites is that the metabolites in urine represent the final state of metabolism, while the metabolites in blood may continue to participate in metabolism. To date, the data of urine metabolomics on the potential hepatotoxicity of aurantio-obtusin in normal rats are still lacking. In this study, the nontargeted metabolomics method was used to study the dynamic changes of urinary metabolite profile and metabolic pathway in the control group and aurantio-obtusin group based on UPLC-QTOF/MS. In our study, 23 metabolites were distributed in 9 metabolic pathways and identified as potential biomarkers of aurantio-obtusin in urine. Among the 23 identified biomarkers, 13 were down-regulated in the urine of rats in aurantio-obtusin group, and the other 10 were up-regulated. Most of the metabolites were directly or indirectly linked to each other, and these biomarkers did not directly affect the citrate cycle (TCA cycle). However, the TCA cycle function can be viewed as a bridge connected with other disturbed metabolic pathways. The TCA cycle is an important aerobic pathway for the final steps of the oxidation of carbohydrates and fatty acids. The TCA cycle is the final metabolic pathway of three major nutrients (carbohydrates, lipids, and amino acids), and also a central role in the metabolism of carbohydrates, fatty acids, amino acids, and so on. For example, succinate is an intermediate in TCA cycle, at the same time it is in phenylalanine metabolism pathway. Therefore, we construct the metabolic network of these 23 biomarkers with the TCA cycle as a center in this paper (**Figure 8**).

### Amino Acids Metabolism

The concentration of amino acids is usually stable with little fluctuation in urine of normal rats. When the liver damage occurs, the levels of amino acids are disordered, breaking the balance between protein and amino acids (synthesis and decomposition) in the organism. Guanidoacetic acid, a water-soluble uremic toxin, is synthesized from arginine and glycine by glycine amidinotransferase. It is transferred to the liver to form a creatine in the reaction catalyzed by guanidinoacetate methyltransferase (GAMT) (Jovanov et al., 2018). It has important biological roles, including the effect on Na^+^, K^+^-ATPase enzyme activity, and GABA receptor. *In vitro*, it was one of cytokines associated with inflammation (Glorieux et al., 2004). It is a metabolite in the metabolic pathways of several amino acids, such as glycine, serine, threonine, arginine, and proline metabolism (Duranton et al., 2012). Compared with the control group, the level of guanidoacetic acid decreased obviously in the aurantio-obtusin treated groups, suggesting that these metabolic pathways were perturbed and might be related to hepatotoxicity induced by aurantio-obtusin. Meanwhile, we observed the down-regulation of 3-methyldioxyinole and 4,6-dihydroxyquinoline in the urine of aurantio-obtusin treated group. They are involved in the tryptophan metabolism. 3-Methyldioxyindole is an oxidation product of 3-methylindole *in vivo* which is a metabolic product of tryptophan, and produced by bacteria in the colon. In the urine of a patient with acute coronary syndrome, 3-methyldioxyindole was found to be up-regulated as a potential metabolic biomarker (Wang et al., 2018). Zhao et al. reported that an obvious decrease of 3-methyldioxyindole was observed in chronic kidney disease rats (Zhao et al., 2013). In this study, it was speculated that aurantio-obtusin interfered with the oxidation process of 3-methylindole, resulting in the down-regulation of 3-methylioxyindole level. It might be a molecular explanation for the hepatotoxicity of aurantio-obtusin, and the relationship between aurantio-obtusin and gut flora needed further study. 4,6-Dihydroxyquinoline is the product of the conversion of 5-hydroxykynurenamine by the enzyme monoamine oxidase. The anti-hyperlipidemia treatment of aloe emodin was reported by Ji, which suggested 4,6-dihydroxyquinoline was downregulated as a biomarker in urine (Ji et al., 2018). This study suggested that the mechanism of lowering blood lipid of aurantio-obtusin might be related to the downregulation of 4,6-dihydroxyquinoline level. Xanthurenic acid is a substrate of the enzyme methyltransferases in tryptophan metabolism. Urinary xanthine acid excretion is an indicator of pyridoxine deficiency and leads to a disorder of tryptophan metabolism (Ulvik et al., 2020). Up-regulation of xanthurenic acid in the high dosage group indicated aurantio-obtusin could generate the dysfunction of tryptophan metabolism in aurantio-obtusin-induced liver injury rats. The earlier study suggested that hepatic injury might be related to metabolic disorders in tryptophan metabolism (Ogihara et al., 1966). 5-L-Glutamyl-taurine is an intermediate in taurine and hypotaurine metabolism, and is produced from taurine *via* the enzyme gamma-glutamyltranspeptidase. Aurantio-obtusin might induce the activity of enzyme gamma-glutamyltranspeptidase and increase the biosynthesis of 5-L-glutamyl-taurine. The increased activity of enzyme gamma glutamyltranspeptidase can lead to various types of liver damage (Meister et al., 2015). In addition, we observed that the decrease of homocitric acid and hydroquinone level and the increase of hippuric acid level in the urine of aurantio-obtusin group. Homocitric acid is a normal urinary organic acid and is involved in lysine biosynthesis (Boulat et al., 2003). It is formed by homocitrate synthase due to propionyl-CoA carboxylase deficiency. Homocitrate synthase is considered as a novel therapeutic target of immunologic deficiency syndromes (Sastoque et al., 2020). This study speculated that aurantio-obtusin interfered with homocitric acid by affecting homocitrate synthase, but the relationship between aurantio-obtusin and immune deficiency needed further study. Hydroquinone, also known as benzene-1, 4-diol, is an aromatic organic compound. However, it can cause toxicity in several organs such as liver, kidney, and nervous system (Topping et al., 2007; Bahadar et al., 2015). The hepatotoxicity induced by aurantio-obtusin may be influenced by the change of hydroquinone in tyrosine metabolism disorder, which also occurred in the previous study of serum metabolomics. Hippuric acid is an acylglycine formed by the conjugation of benzoic acid with glycine. Existing studies have demonstrated that chronic kidney disease is related to the accumulation of hippuric acid (Velenosi et al., 2016). The dynamical growing of hippuric acid maybe was resulted from the continuous impact of microbial metabolic disorders. In this study, it indicated hippuric acid could be considered as a biomarker for aurantio-obtusin nephrotoxicity and cause the disorder of phenylalanine metabolism which was produced by gut microflora. To date, there are no studies on aurantio-obtusin nephrotoxicity, which needs further verification.

### Bile Acid Metabolism

Bile acids are steroid carboxylic acids derived from cholesterol in vertebrates. Cholic acid is one of the primary bile acids produced by cholesterol in the liver (Petrov et al., 2020). Glycocholic acid is a secondary metabolic bile acid produced by gut flora (Heubi et al., 2015). Their contents were all downregulated in the urine of aurantio-obtusin group, which might be the liver damage caused by aurantio-obtusin. Alternatively, bile acids are reabsorbed back to the liver in the ileum and colon and deposited in the liver so that they cannot be excreted in urine through the kidney. In our previous study of serum metabolomics, it also concluded that aurantio-obtusin could cause the disorder of bile acid metabolism in rats. In summary, aurantio-obtusin could cause the abnormal metabolism of bile acid and the abnormal enterohepatic circulation in rats.

### Carbohydrate Metabolism

Carbohydrate metabolism is one of the most important biological processes in organism metabolisms. Both glyoxylate and dicarboxylate metabolism and ascorbate and aldarate metabolism belong to carbohydrate metabolism. Hydroxypyruvic acid is a substrate for serine pyruvate aminotransferase, glyoxylate reductase, and hydroxypyruvate reductase. It has a role as a human metabolite and an Escherichia coli metabolite. Hydroxypyruvic acid is a metabolite of serine, which may induce renal injury (Williams et al., 2005), so it needs to be further explored the effect of upregulated hydroxypyruvic acid on hepatorenal toxicity. Ascorbic acid can reduce cholesterol through poorly described mechanisms, or improve vasodilation and vascular reactivity by reducing the interaction of nitric oxide with oxidants (Padayatty and Levine, 2000). Rudra et al. reported that the concentrations of ascorbic acid were increased significantly in the liver tissues and in the urine of the toxicated rats (Rudra et al., 1975). Indeed, ascorbic acid is protective against hepatotoxic substances and provides antioxidant and cytoprotective activity to hepatocytes in many animal models, but higher doses of ascorbic acid have been reported to result in serum alanine transaminase (ALT) elevations. Up-regulation of ascorbic acid may be related to the interference of aurantio-obtusin on carbohydrate in animals, affecting the citric acid cycle, which is the final metabolic pathway of three major nutrients: carbohydrate, lipid, and amino acid. Therefore, these findings indicated that ascorbate and aldarate metabolism was associated with the pathogenesis of hepatotoxicity.

### Nucleotide Metabolism

The liver plays a central role in systemic nucleoside homeostasis *in vivo*. Hepatocytes have the capacity for *de novo* synthesis of both purine and pyrimidine nucleotides (Zhang et al., 2019). The pathway of decomposition also occurs in the liver. DADP is a purine 2’-deoxyribonucleoside 5’-diphosphate having adenine as the nucleobase. DADP is formed during purine catabolism as a product of hypoxanthine oxidase action on hypoxanthine, and hypoxanthine can be metabolized to xanthosine (Simmonds et al., 1982). Xanthosine is an intermediate in the degradation of adenosine monophosphate to uric acid, being formed by oxidation of hypoxanthine (Dong et al., 2020). In this study, the intensity of xanthosine and dADP significantly increased in aurantio-obtusin group compared to the control group, indicating that purine metabolism was altered by aurantio-obtusin. Thymidine is a pyrimidine nucleoside and derives from a thymine. Its decrease indicated the disorder of pyrimidine metabolism (Shaw and Locarnini, 1995). The imbalance of purine and pyrimidine metabolism may be due to the damage of aurantio-obtusin to liver.

### Others

Finally, we also found some metabolites involved in other metabolic pathways with significant changes. Sebacic acid is a normal urinary acid, and is involved in lipid and fatty acid metabolism. Some studies have shown that sebacic acid can be used as a marker of hepatorenal toxicity in urine of rats (Han et al., 2016). 5-Hydroxysebacate is a metabolite found in the urine of patients with peroxismal diseases. The decrease of sebacic acid and 5-hydroxysebacate indicated that aurantio-obtusin disturbed the oxidative function of rats, which were harmful to the liver and kidney. Indoxyl sulfate and phenol have been identified as a uremic toxin according to the European Uremic Toxin Working Group, which can damage the liver and kidney. Compared with the control group, the level of indoxyl sulfate and phenol increased obviously in the aurantio-obtusin treated groups. Menadione can be converted to active vitamin K2, menaquinone, after alkylation *in vivo*. Large doses of menadione have been reported to cause liver damage (Badr et al., 1989). The findings show that menadione induces testicular toxicity by depleting the antioxidant defense system (Balogun et al., 2019). An obvious increase of menadione was observed in aurantio-obtusin rats in this study.

Nutriacholic acid is a bile acid. Bile acids are physiological detergents that facilitate excretion, absorption, and transport of fats and sterols in the intestine and liver. Nutriacholic acid is used as a prognostic metabolite in the study of predicting tumor recurrence after liver transplantation (Lu et al., 2019). Down-regulation of nutriacholic acid might be induced by aurantio-obtusin, which would cause the abnormality of bile acid metabolism directly. In addition, down-regulation of 6-methylmercaptopurine and tetrahydroaldosterone-3-glucuronide were observed in the aurantio-obtusin treated rats. It has been reported that thiopurines can cause cytolytic and cholestatic liver injury and liver toxicity appears to be related to high methylated derivative levels, mainly 6-methylmercaptopurine (Nygaard et al., 2004). Tetrahydroaldosterone-3-glucuronide is a natural human metabolite of tetrahydroaldosterone generated in the liver by uridine 5’-diphospho (UDP)-glucuronosyltransferase. The decrease might be due to the liver damage caused by high dose of aurantio-obtusin, which affected the activity of UDP glucosyltransferase. Marks et al. demonstrated it was associated with a reduction in blood pressure that the diminished formation of the metabolite tetrahydroaldosterone-3-glucuronide (Marks et al., 1985). This was the competitive inhibition of glucuronidase by aurantio-obtusin (Yu et al., 2014), which provided a mechanism for aurantio-obtusin to reduce blood pressure.

Conclusively, the biomarkers revealed in this paper are useful in providing clues for further research on the toxicity and efficacy of aurantio-obtusin.

## Conclusion

In this study, a method of urinary metabolomics of aurantio-obtusin was established based on the technology of UPLC-QTOF/MS. The current study demonstrated that the urine metabolites profiles were significantly different between the control and aurantio-obtusin-treated rats. Our study confirmed that there were 23 potential biomarkers with significant changes, which were mainly enriched in purine metabolism, taurine and hypotaurine metabolism, primary bile acid biosynthesis, pyrimidine metabolism, and tryptophan metabolism, suggesting that they might be an important way of aurantio-obtusin-induced liver injury.

This is the first discovery that endogenous biomarkers are associated with aurantio-obtusin-induced liver injury in urine, and these biomarkers can be used as important early diagnostic indicators for the potential hepatotoxicity of aurantio-obtusin. Meanwhile, it may provide a clue for the evaluation of renal toxicity of aurantio-obtusin.

## Data Availability Statement

All datasets presented in this study are included in the article/**Supplementary Material**.

## Ethics Statement

The animal study was reviewed and approved by Animal Care and Use Committee, Academy of Military Medical Sciences, Beijing, China.

## Author Contributions

LX performed the experiments, analyzed the data and wrote the manuscript. XT, YW, and ZM performed the experiments. YG designed the experiment and edited the manuscript, and is the guarantor of this study. All authors contributed to the article and approved the submitted version.

## Funding

This work was supported by the National Natural Science Foundation of China [Nos. 81630102 and 81673633] and the National Science and Technology Major Project of China [No. 2015ZX09501004-003-003] and the National Key Research and Development Project of China [No.2019YFC1604900].

## Conflict of Interest

The authors declare that the research was conducted in the absence of any commercial or financial relationships that could be construed as a potential conflict of interest.
