# Supplementary material for: Urine Metabolomics Study on Potential Hepatoxic Biomarkers Identification in Rats Induced by Aurantio-Obtusin
Source: Front Pharmacol. 2020 Aug 12;11:1237. doi: 10.3389/fphar.2020.01237 (PMC7435054; doi:10.3389/fphar.2020.01237)
Supplement: Supplementary file 1 [file DataSheet_1.docx]

Supplementary Material

# Abbreviations

Abbreviations: UPLC-QTOF/MS = Ultra Performance Liquid Chromatography Quadrupole Time-of-Flight Mass Spectrometry, ESI = electrospray ionization, FC = fold change, KEGG = Kyoto Encyclopedia of Genes and Genomes, HMDB = Human Metabolome Database, PCA = principal component analysis, PLS-DA = partial least squares discriminant analysis, OPLS-DA =orthogonal projection to latent structures squares-discriminant analysis, QC = quality control, RT = retention time, VIP = variable importance in projection, m/z = mass to nucleus ratio, EIC = extracted ion chromatogram.

# Supplementary Tables

Table S1 Conditions of UPLC gradient elution.

| Time(min) | Flow rate | A/%(0.1% | B/(0.1% | Curve |
| --- | --- | --- | --- | --- |
|  | (mL/min) | formic H_2_O | formic ACN |  |
| Initial | 0.5 | 98 | 2 | – |
| 0.5 | 0.5 | 98 | 2 | 6 |
| 10 | 0.5 | 70 | 30 | 6 |
| 12 | 0.5 | 2 | 98 | 6 |
| 13 | 0.5 | 98 | 2 | 1 |
| 15 | 0.5 | 98 | 2 | 6 |

Table S2 The relative standard deviations (RSD) of T_R_ and the peak area in positive and negative mode.

| Ion Mode | T_R__m/z | RSD(%) | |
| --- | --- | --- | --- |
|  |  | T_R_ | Area |
| Positive | 1.20_136.0454 | 0 | 7.01 |
|  | 3.95_372.2416 | 0 | 3.6 |
|  | 6.72_170.0664 | 0 | 2.59 |
|  | 8.15_331.0855 | 0.28 | 0.78 |
|  | 12.06_284.3367 | 0 | 7.67 |
| Negative | 2.30_188.9866 | 0 | 2.28 |
|  | 3.76_212.0033 | 0 | 1.42 |
|  | 5.34_283.0839 | 0 | 6.16 |
|  | 9.03_343.0854 | 0 | 3.49 |
|  | 11.19_329.0671 | 0 | 7.07 |

Table S3 Parameters of PCA, PLS-DA and OPLS-DA models.

| Mode | Model | Component | R2X(cum) | R2Y(cum) | Q2(cum) | R2-intercept | Q2-intercept |
| --- | --- | --- | --- | --- | --- | --- | --- |
| ESI+ | PCA | 5 | 0.693 | — | 0.468 | — | — |
|  | PLS-DA | 5 | 0.749 | 0.834 | 0.569 | 0.126 | -0.143 |
|  | OPLS-DA | 2 | 0.673 | 0.977 | 0.957 | — | — |
| ESI- | PCA | 5 | 0.653 | — | 0.443 | — | — |
|  | PLS-DA | 5 | 0.707 | 0.947 | 0.612 | 0.373 | -0.364 |
|  | OPLS-DA | 2 | 0.63 | 0.995 | 0.966 | — | — |

R2-intercept and Q2-intercept represent result of permutation tests with 200 iterations.

Table S4 Potential biomarkers identified among high dose group with the control group in positive and negative ESI mode.

| No. | ESI mode | R.T.(min) | m/z found | ppm | Formula | Identifier | KEGG | HMDB | Fold change | P value | VIP | Trend | Identification method | Related pathway |
| --- | --- | --- | --- | --- | --- | --- | --- | --- | --- | --- | --- | --- | --- | --- |
| 1 | M+H | 3.73 | 118.0706 | 10.0 | C_3_H_7_N_3_O_2_ | Guanidoacetic acid | C00581 | 0000128 | 3.7 | 0.0428 | 3.6 | Down | Standard | Arginine and proline metabolism |
| 2 | M+Na | 12.51 | 413.2690 | 7.0 | C_24_H_38_O_4_ | Nutriacholic acid | Unknown | 0000467 | 11.6 | 0.0435 | 3.9 | Down | Standard | Unknown |
| 3 | M+H | 12.33 | 167.0395 | 5.0 | C_6_H_6_N_4_S | 6-Methylmercaptopurine | C16614 | 0060412 | 14.8 | 0.0002 | 4.8 | Down | Database | Drug metabolism |
| 4 | M+H-H2O | 12.36 | 391.2864 | 4.0 | C_24_H_40_O_5_ | Cholic acid | C00695 | 0000619 | 48.3 | 0.0004 | 15.9 | Down | Standard | Primary bile acid biosynthesis |
| 5 | M+H | 6.14 | 243.0959 | 7.0 | C_10_H_14_N_2_O_5_ | Thymidine | C00214 | 0000273 | 2.6 | 0.0005 | 3.0 | Down | Standard | Pyrimidine metabolism |
| 6 | M+H | 7.24 | 285.0807 | 8.0 | C_10_H_12_N_4_O_6_ | Xanthosine | C01762 | 0000299 | 1.7 | 0.0002 | 2.7 | Up | Standard | Purine metabolism |
| 7 | M+H | 2.85 | 541.2605 | 7.0 | C_27_H_40_O_11_ | Tetrahydroaldosterone-3-glucuronide | Unknown | 0010357 | 8.2 | 0.0207 | 3.3 | Down | Database | Unknown |
| 8 | M-H | 4.49 | 162.0571 | 6.0 | C_9_H_9_NO_2_ | 3-Methyldioxyindole | C05834 | 0004186 | 13.3 | 0.0003 | 7.2 | Down | Database | Tryptophan metabolism |
| 9 | M-H | 9.65 | 410.0268 | 1.0 | C_10_H_15_N_5_O_9_P_2_ | dADP | C00206 | 0001508 | 2.2 | 0.0005 | 6.4 | Up | Database | Purine metabolism |
| 10 | M-H | 8.70 | 201.1140 | 4.0 | C_10_H_18_O_4_ | Sebacic acid | C08277 | 0000792 | 2.8 | 6.17E-08 | 6.4 | Down | Standard | Unknown |
| 11 | M-H | 3.76 | 178.0510 | 0.0 | C_9_H_9_NO_3_ | Hippuric acid | C01586 | 0000714 | 1.9 | 3.85E-06 | 6.7 | Up | Standard | Phenylalanine metabolism |
| 12 | M-H | 12.72 | 204.0312 | 5.0 | C_10_H_7_NO_4_ | Xanthurenic acid | C02470 | 0000881 | 29.5 | 0.0006 | 7.9 | Up | Standard | Tryptophan metabolism |
| 13 | M-H | 4.13 | 160.0409 | 3.0 | C_9_H_7_NO_2_ | 4,6-Dihydroxyquinoline | C05639 | 0004077 | 1.6 | 0.0140 | 3.4 | Down | Database | Tryptophan metabolism |
| 14 | M-H | 2.28 | 109.0297 | 2.0 | C_6_H_6_O_2_ | Hydroquinone | C00530 | 0002434 | 23.5 | 0.0158 | 3.6 | Down | Database | Tyrosine metabolism |
| 15 | M-H | 8.12 | 175.0258 | 3.0 | C_6_H_8_O_6_ | Ascorbic acid | C01041 | 0000044 | 2.2 | 3.05E-05 | 3.2 | Up | Standard | Ascorbate and aldarate metabolism |
| 16 | M-H | 5.56 | 217.1084 | 1.0 | C_10_H_18_O_5_ | 5-Hydroxysebacate | Unknown | 0029189 | 2.0 | 0.0001 | 2.9 | Down | Database | Unknown |
| 17 | M-H | 7.17 | 253.0516 | 6.0 | C_7_H_14_N_2_O_6_S | 5-L-Glutamyl-taurine | C05844 | 0004195 | 1.5 | 0.0241 | 2.7 | Up | Database | Taurine and hypotaurine metabolism |
| 18 | M-H | 2.70 | 93.0350 | 4.0 | C_6_H_6_O | Phenol | C15584 | 0000228 | 1.6 | 0.0002 | 4.5 | Up | Database | Unknown |
| 19 | M-H | 3.75 | 212.0031 | 4.0 | C_8_H_7_NO_4_S | Indoxyl sulfate | Unknown | 0000682 | 2.2 | 0.0007 | 2.2 | Up | Database | Unknown |
| 20 | M-H | 8.10 | 103.0045 | 8.0 | C_3_H_4_O_4_ | Hydroxypyruvic acid | C00168 | 0001352 | 6.3 | 2.53E-06 | 4.1 | Up | Database | Glyoxylate and dicarboxylate metabolism |
| 21 | M-H | 9.61 | 171.0459 | 4.0 | C_11_H_8_O_2_ | Menadione | C05377 | 0001892 | 3.6 | 0.0005 | 3.8 | Up | Standard | Vitamin digestion and absorption |
| 22 | M-H | 2.85 | 205.0346 | 4.0 | C_7_H_10_O_7_ | Homocitric acid | C01251 | 0003518 | 2.1 | 0.0365 | 2.5 | Down | Database | Lysine biosynthesis |
| 23 | M-H | 5.04 | 464.3012 | 1.0 | C_26_H_43_NO_6_ | Glycocholic acid | C01921 | 0000138 | 2.4 | 0.0002 | 4.9 | Down | Standard | Primary bile acid biosynthesis |

Fold change was calculated from the arithmetic mean values of each group.

ppm (Delta) = (abs (query mass – adduct mass) / adduct mass)* 1000000

Table S5 Result from pathways analysis with MetaboAnalyst 4.0.

| Pathway name | Total | Expected | Hits | Raw p | -log(p) | Impact |
| --- | --- | --- | --- | --- | --- | --- |
| Glyoxylate and dicarboxylate metabolism | 32 | 0.3817 | 1 | 5.4E-13 | 28.25 | 0.2196 |
| Phenylalanine metabolism | 12 | 0.1431 | 1 | 4.6E-12 | 26.12 | 0.0000 |
| Glycine, serine and threonine metabolism | 34 | 0.4056 | 2 | 3.7E-11 | 24.03 | 0.0666 |
| Purine metabolism | 66 | 0.7873 | 2 | 1.1E-09 | 20.59 | 0.0095 |
| Taurine and hypotaurine metabolism | 8 | 0.0954 | 1 | 3.6E-08 | 17.14 | 0.0000 |
| Primary bile acid biosynthesis | 46 | 0.5487 | 2 | 1.1E-06 | 13.70 | 0.0229 |
| Arginine and proline metabolism | 38 | 0.4533 | 1 | 6.2E-06 | 11.99 | 0.0235 |
| Pyrimidine metabolism | 39 | 0.4652 | 1 | 0.00012 | 8.99 | 0.0599 |
| Tryptophan metabolism | 41 | 0.4891 | 1 | 0.00853 | 4.76 | 0.0000 |

Total is the total number of compounds in the pathway; the Hits is the actually matched

number from the user uploaded data; the Raw p is the original p value calculated from the

enrichment analysis; the Impact is the pathway impact value calculated from pathway topology

analysis

# Supplementary Figures





FIGURE S1 Chemical structure of anthraquinone, emodin and aurantio-obtusin.


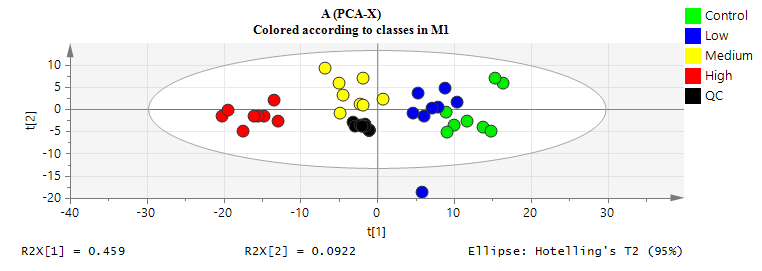


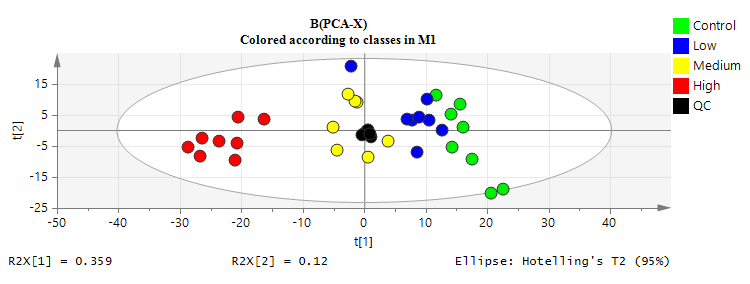


FIGURE S2 PCA score plots base on the data from positive (A) and negative (B) ionization model (the aurantio-obtusin group is shown in blue, yellow and red. The control group is shown in green and the QC group is shown in black.)


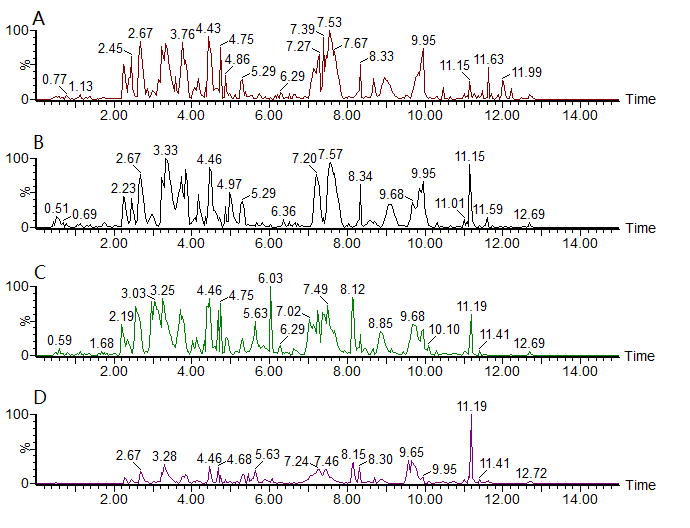


FIGURE S3. The UPLC-QTOF/MS BPI profile of (A) control group, (B) low dosage group, (C) medium dosage group and (D) high dosage group urinary samples on the 28th day in negative ESI mode. Time unit is minute.








FIGURE S4. The UPLC-QTOF/MS EIC profile of high dosage group in (A) positive and (B) negative ESI mode. Peak 1 for guanidoacetic acid, peak 2 for nutriacholic acid, peak 3 for 6-Methylmercaptopurine, peak 4 for cholic acid, peak 5 for thymidine, peak 6 for xanthosine, peak 7 for tetrahydroaldosterone-3-glucuronide, peak 8 for 3-Methyldioxyindole, peak 9 for dADP, peak 10 for sebacic acid, peak 11 for hippuric acid, peak 12 for xanthurenic acid, peak 13 for 4,6-Dihydroxyquinoline, peak 14 for hydroquinone, peak 15 for ascorbic acid, peak 16 for 5-Hydroxysebacate, peak 17 for 5-L-Glutamyl-taurine, peak 18 for phenol, peak 19 for indoxyl sulfate, peak 20 for hydroxypyruvic acid, peak 21 for menadione, peak 22 for homocitric acid and peak 23 for glycocholic acid, respectively.


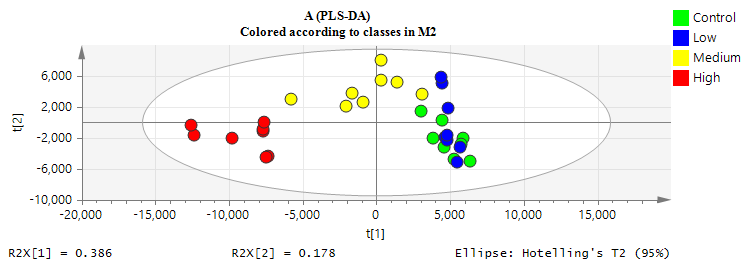


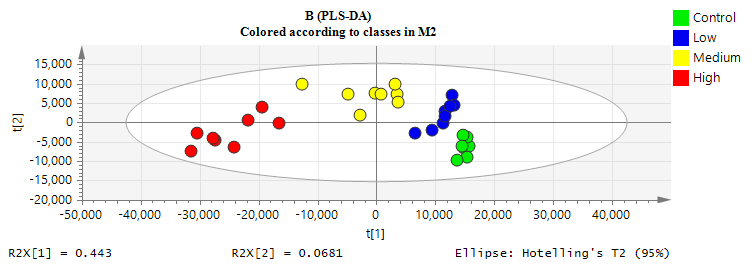


FIGURE S5. PLS-DA score plots of urinary samples collected from control group, low dosage group, medium dosage group and high dosage group in positive ESI mode (A) and negative ESI mode (B).


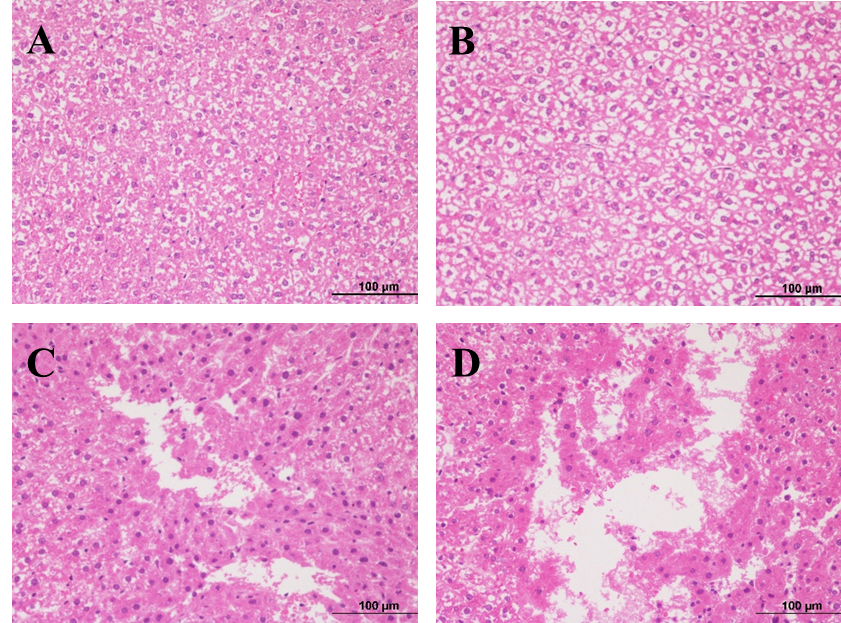


FIGURE S6 The histopathological observation of rat liver by different Aurantio-obtusin dose and stained with hematoxylin and eosin (H & E): A, control group; B, low group; C, medium group; and D, high group.


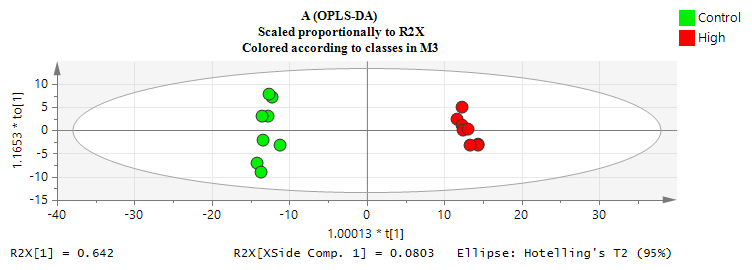


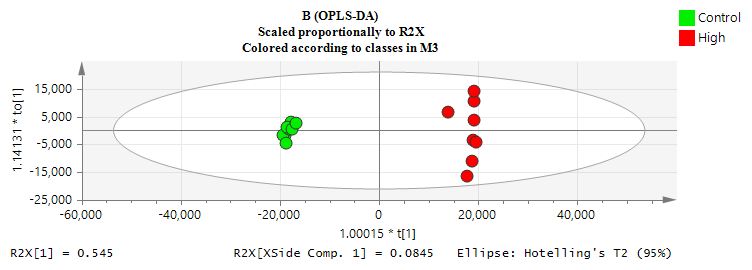


FIGURE S7 OPLS-DA score plots of urinary samples collected from control group, low dosage group, medium dosage group and high dosage group in positive ESI mode (A) and negative ESI mode (B).


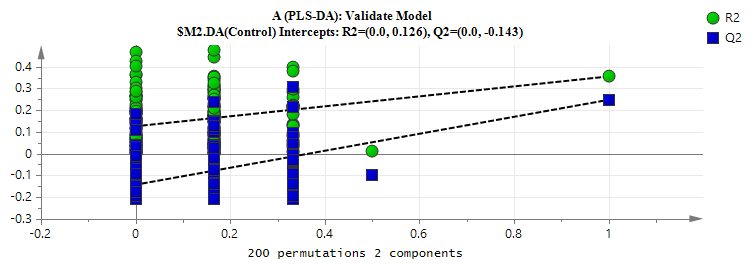


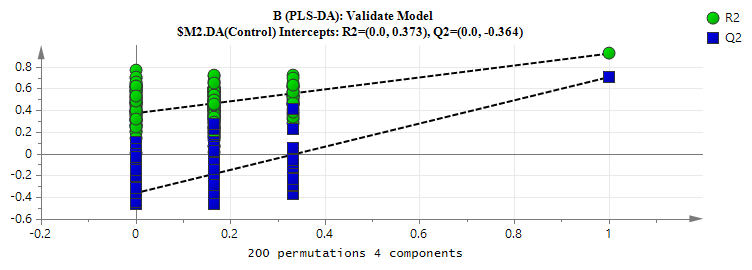


FIGURE S8 Permutation test with 200 iterations of PLS-DA model in positive ESI mode (A) and negative ESI mode (B).








FIGURE S9 Mass fragment information of hippuric acid standard and ion with *m/z*=178.0510 in urine.


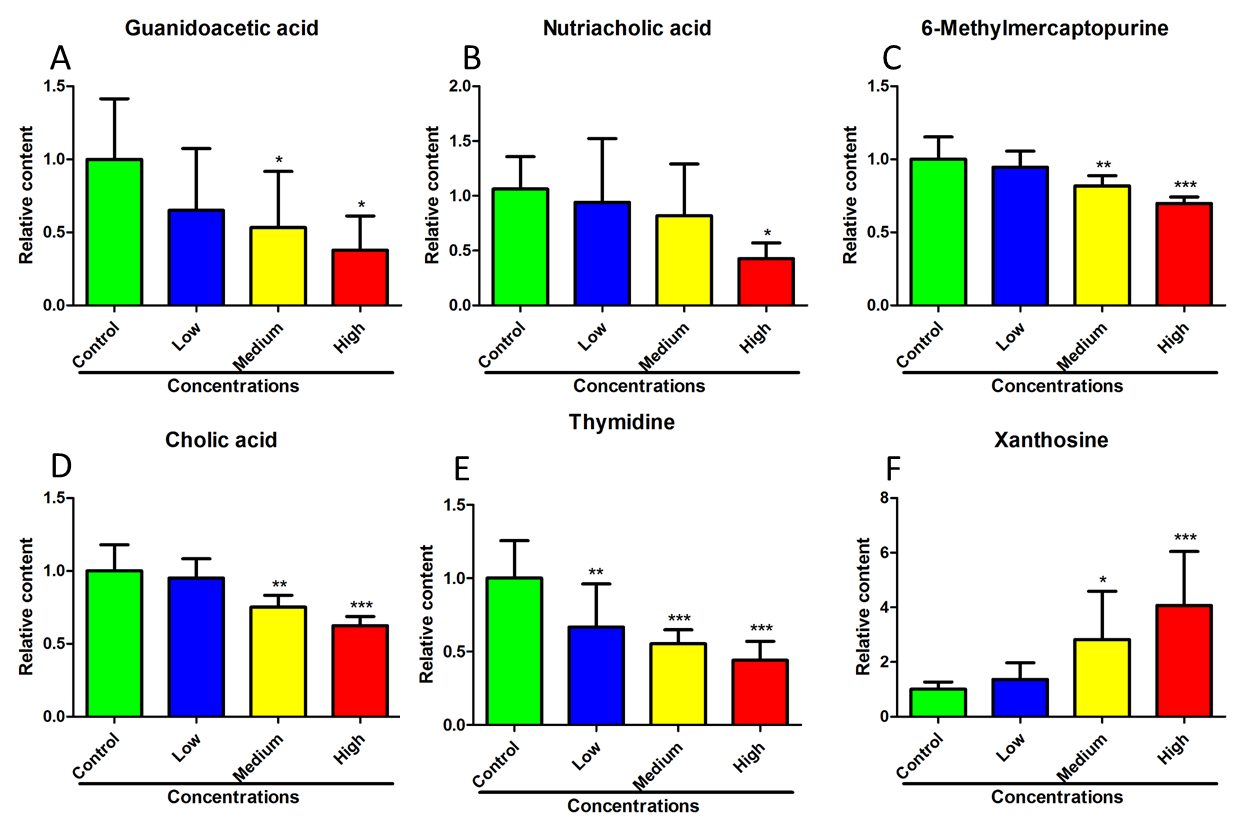


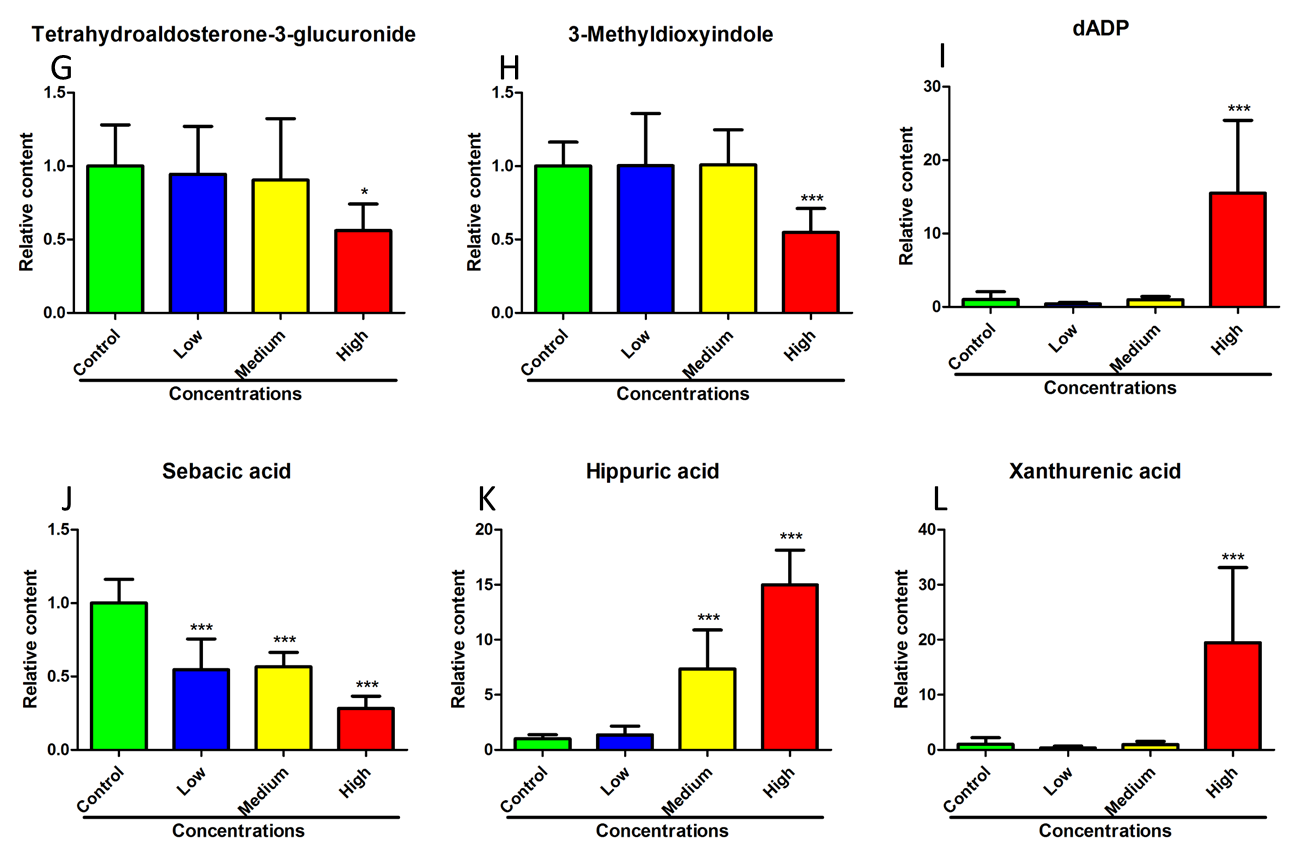


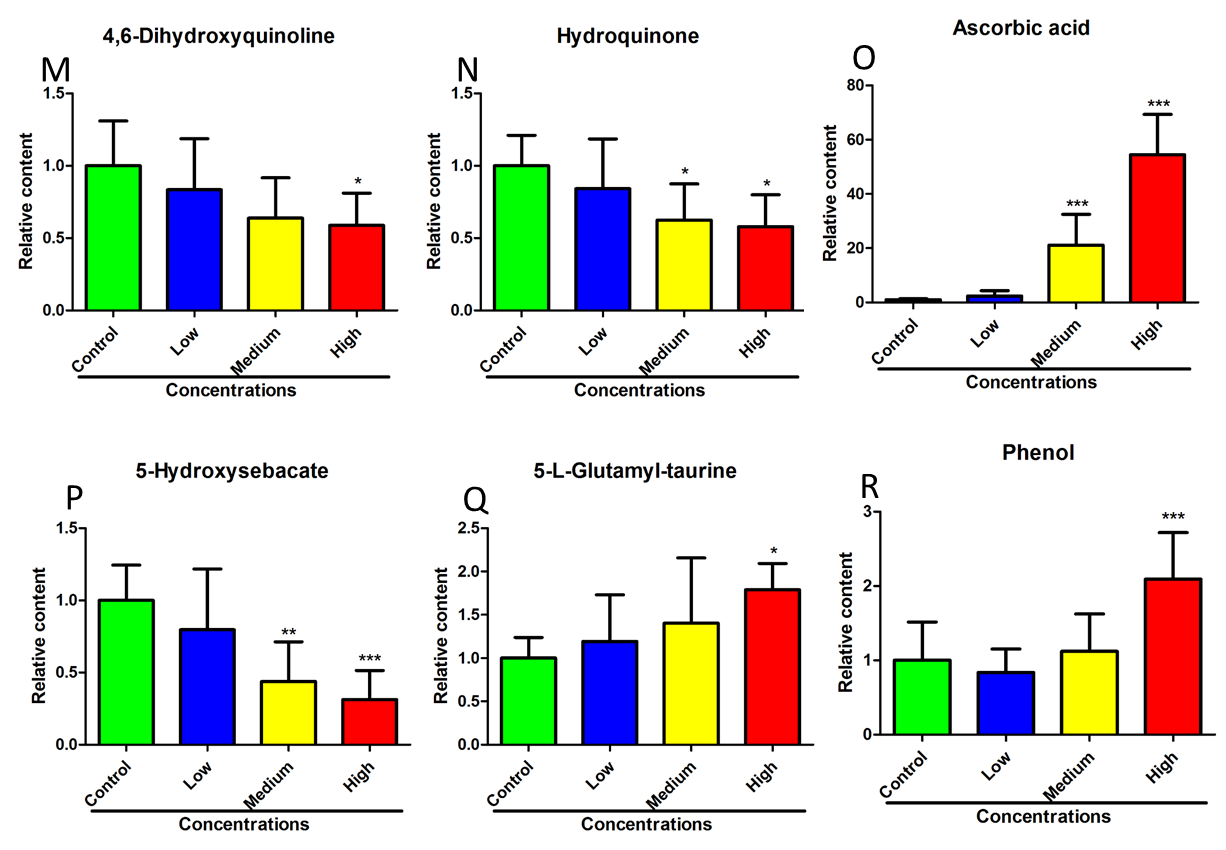


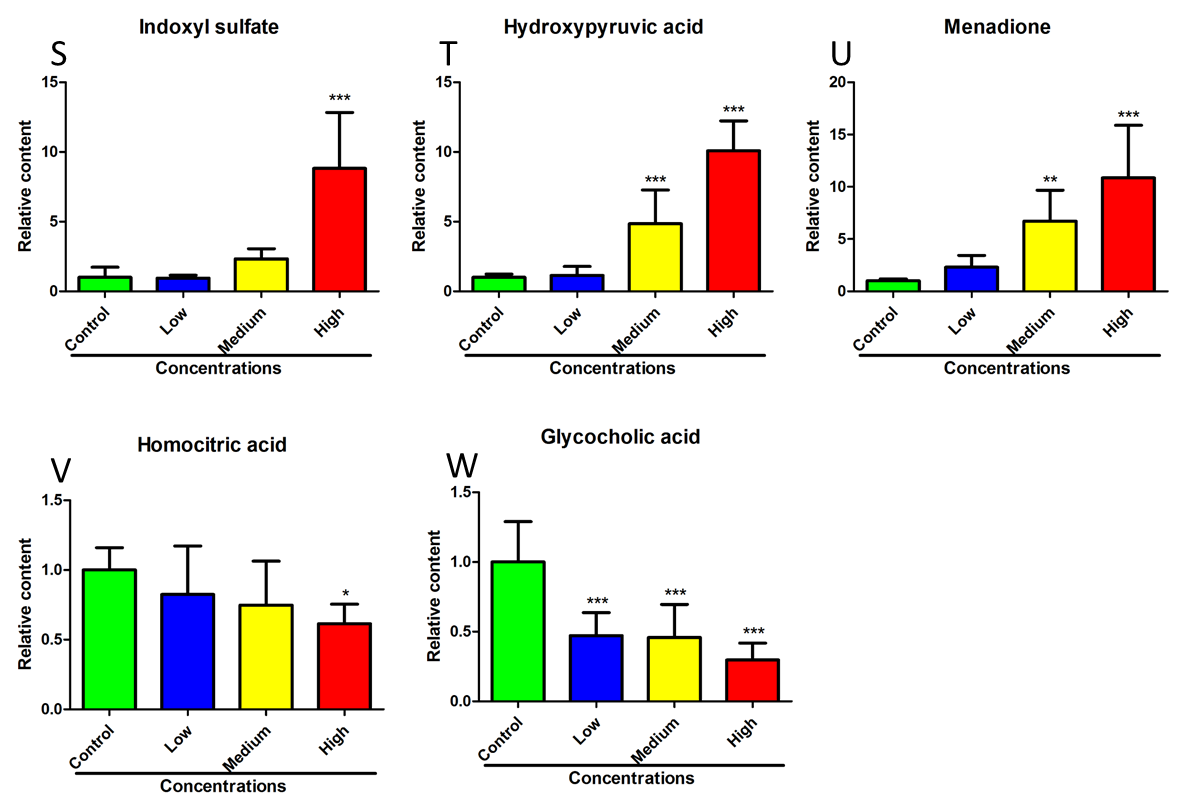


FIGURE S10 Variations in the trends of the metabolites that are biomarkers. (A–W) shown are the variations in the trends of guanidoacetic acid, nutriacholic acid, 6-Methylmercaptopurine, cholic acid, thymidine, xanthosine, tetrahydroaldosterone-3-glucuronide, 3-Methyldioxyindole, dADP, sebacic acid, hippuric acid, xanthurenic acid, 4,6-Dihydroxyquinoline, hydroquinone, ascorbic acid, 5-Hydroxysebacate, 5-L-Glutamyl-taurine, phenol, indoxyl sulfate, hydroxypyruvic acid, menadione, homocitric acid and glycocholic acid, respectively. *P < 0.05, **P < 0.01, ***P<0.001 compared with the control group, respectively.
